# Lung cancer screening among people with HIV, 2018-2023

**DOI:** 10.1177/09564624261469254

**Published:** 2026-07-30

**Authors:** Subhashini A. Sellers, Lindsay E. Browne, Heather I. Henderson, Amanda E. Moy, Deana M. Agil, Amy Durr, Claire E. Farel, Joseph J. Eron, Sonia Napravnik

**Affiliations:** 1 Division of Pulmonary Diseases and Critical Care Medicine, University of North Carolina at Chapel Hill, Chapel Hill, NC, USA; 2 Division of Infectious Diseases, University of North Carolina at Chapel Hill, Chapel Hill, NC, USA

**Keywords:** lung cancer, screening, HIV, clinical care

## Abstract

**Background:**

Lung cancer is the leading cause of cancer-related death among persons with HIV (PWH) in the United States. Annual screening with low-dose chest computed tomography may reduce mortality, but uptake remains unclear. We sought to characterize lung cancer screening eligibility and uptake among PWH engaged in HIV care at a Southeastern tertiary care center.

**Methods:**

A cross-sectional analysis with 6 time points corresponding to each calendar year from 2018 through 2023 among PWH receiving care. Data were collected from electronic health records, clinical event adjudications, and patient-reported outcomes. Prevalence differences and 95% CIs were estimated across demographic and clinical characteristics.

**Results:**

Among 2798 PWH in care, 74% were men, 57% identified as non-Hispanic Black and the median age was 48 years (IQR: 35-57). Among all PWH, 62% had a history of smoking with a median pack-year history of 40 (IQR: 27-50) and 51% of these were current smokers. The proportion of PWH meeting United States Preventative Services Task Force screening criteria increased from 6% in 2018 to 15% by 2023 (*p* < 0.05). Overall, 13% of PWH were eligible for screening, among whom less than one-third (27%) received screening at least once during follow-up. Annual screening was 11% and 24% in 2018 and 2023, respectively, (*p* = 0.10).

**Conclusions:**

Lung cancer screening utilization remains low, even among a high-risk population in the Southeastern United States. Understanding barriers to screening is needed to improve lung cancer outcomes among PWH.

## Introduction

Lung cancer is the most common cause of malignancy-related death and a leading cause of overall mortality among persons living with HIV (PWH) in the United States.^1–3^ This is due in part to the prevalence of smoking in PWH, which is twice as high as in the general population.^4^ Additionally, HIV infection itself is independently associated with lung cancer risk, likely mediated by the effect of the virus on airway epithelial cell structure, pulmonary immune alterations, and oxidative stress.^5–10^ Moreover, lung cancer in PWH is often diagnosed at younger ages and with lower cumulative smoking histories,^8–12^ and PWH experience shorter survival compared to people without HIV, even when controlling for cancer stage at diagnosis.^10,13–16^ These factors underscore the urgent need for effective lung cancer prevention and early detection strategies in this population.

Lung cancer screening (LCS) via annual low-dose computed tomography (LDCT) of the chest has been shown to improve lung cancer mortality by up to 20% in the general population.^17^ This evidence served as the basis for the 2013 U.S. Preventive Services Task Force (USPSTF) recommendations supporting annual screening for adults aged 55–80 with a ≥30 pack-year smoking history who currently smoke or have quit within the past 15 years^18^ While PWH were under-represented in the National Lung Screening Trial, projections suggest that implementing these guidelines among PWH could reduce lung cancer mortality by 19%.^19^

In March 2021, the USPSTF updated its recommendations, lowering the eligible age to 50 and the pack-year minimum to 20, thereby expanding eligibility for screening.^20,21^ Our prior work demonstrated that the 2021 guidelines yield higher sensitivity in identifying lung cancer cases among PWH compared with the 2013 recommendations.^22^ Despite anticipated increases in screening eligibility, mortality benefit is predicated on widespread uptake of recommendations, which has been variable in the general population, with estimates from 16 to 47% of eligible individuals undergoing some degree of screening.^23–25^ To the best of our knowledge, uptake of these new recommendations has not been evaluated in PWH who experience unique barriers to care access. This study evaluated the annual prevalence of LCS eligibility and uptake among PWH in a large Southeastern U.S. HIV clinic from 2018 through 2023. Our hypothesis was that uptake of LCS was poor among PWH and that missed opportunities for identifying lung cancer early increased with release of the updated screening criteria.

## Methods

### Study population

We used a cross-sectional study design. Our study population included participants in the University of North Carolina (UNC) Center for AIDS Research HIV Clinical Cohort (UCHCC), which includes over 7000 PWH who have received care in the UNC HIV Clinic since 1996 and has been previously described.^26^ For these analyses our data included electronic health records (e.g., demographics, laboratory values), patient-reported outcomes (e.g., smoking), adjudicated clinical outcomes (e.g., lung cancer), and external data sources (e.g., mortality data from State records and the National Death Index). We included all PWH aged ≥18 with at least one clinic visit between January 1, 2018, and December 31, 2023. PWH provided informed consent to participate in the UCHCC, with most (>96%) agreeing to participate. The present study was approved by the UNC Institutional Review Board.

### Data elements

Demographic and clinical data, including age, sex, race/ethnicity, insurance status, CD4 cell count, and HIV viral load, were extracted from the electronic health record. Rurality was measured using United States Department of Agriculture Rural-Urban Commuting Area codes.^27^ Smoking history was assessed using electronic health records and patient reported outcomes, which were collected approximately every 6 months as part of routine clinical care.^28^ Annual smoking status was categorized as current, former, and never smoker, with smoking intensity calculated as pack-years of cigarette smoking.

### Lung cancer screening

Patients were eligible for LCS based on the 2013 USPSTF guidelines between January 1, 2018 and December 31, 2021, and the 2021 USPSTF guidelines from January 1, 2022 to December 31, 2023.^20,21^ To establish screening we used CPT code 71,271 (introduced January 1, 2021), HCPCS code G0297 (used until December 31, 2020), and HCPCS code S8032 (superseded by G0297, then 71,271).

### Statistical analyses

We estimated the annual prevalence of LCS eligibility and utilization, stratified by demographic and clinical characteristics. Time-varying factors were updated at beginning of each calendar period in care. For annual LCS eligibility, patients contributed to the denominator if they had at least one HIV care appointment and the numerator if they met relevant USPSTF criteria in that calendar year. For each annual estimate, patients were included in the denominator if they met the USPSTF guidelines for screening in that calendar year and in the numerator if they received LCS in that calendar year. Patients with lung cancer were excluded from subsequent eligibility after diagnosis. Unadjusted generalized linear models were used to estimate prevalence differences (PD) and 95% confidence intervals (CI). Chi-square tests were used to compare the proportion of patients eligible for LCS and the proportion of eligible patients receiving LCS before versus after the 2021 USPSTF guideline update.

## Results

### Study population

Between January 1, 2018 and December 31, 2023, 2798 PWH received HIV care and contributed to a median of 5 calendar years of analysis (IQR: 2-6). At start of follow-up the median age was 48 years (IQR: 35-57; range: 18-91), 74% were men, 57% non-Hispanic Black, 28% non-Hispanic White, 9% Hispanic, and 5% other races/ethnicity. Patients were in HIV care a median of 11.0 years (IQR: 4.6-18.7), 76% had HIV RNA levels <200 copies/mL and the median CD4 cell count was 599 cells/mm^3^ (IQR: 374-841). Also at baseline, 1742 (62%) reported any history of tobacco smoking, among whom 51% reported current smoking and 49% former smoking, with a median smoking history of 40 pack-years (IQR: 27–50). Of former smokers, 738 (86%) had quit within 15 years from analysis date.

The study population included N = 2452 in years 2018-2021, and N = 2209 in years 2022-2023 (Table 1). While most demographic and clinical characteristics remained stable over time, the median age of the population increased from 46 years (IQR: 33–55) in 2018–2021 to 51 years (IQR: 37–60) in 2022–2023 (*p* < 0.001). The proportion of patients with HIV RNA <200 copies/mL at the beginning of each interval, which includes the first value after initiating care for some, rose from 79% in 2018–2021 to 86% in 2022–2023 (*p* < 0.001). Notably, the proportion of patients in HIV care for at least 5 years increased from 68% in 2018-2021 to 83% in 2022-2023 (*p* < 0.001).

**Table 1.** Patient characteristics and lung cancer screening eligibility, stratified by calendar year, 2018-2023.

Characteristic^b^	Calendar years			
	2018-2021		2022-2023	
	All (N, %)	Eligible^a^ (%)	All (N, %)	Eligible^a^ (%)
Overall	2452	6	2209	15
Age (years), median (IQR)	46 (33 - 55)	​	51 (37 - 60)	​
18–49	1451 (59%)	0	1060 (48%)	1
50–59	678 (28%)	14	608 (28%)	27
60–69	267 (11%)	20	419 (19%)	30
70–80	46 (2%)	13	110 (5%)	26
81+	10 (0%)	0	12 (1%)	0
Sex				
Female	652 (27%)	7	591 (27%)	17
Male	1800 (73%)	6	1618 (71%)	14
Race/ethnicity				
Black	1417 (58%)	5	1274 (58%)	14
Hispanic or other^c^	345 (14%)	1	315 (14%)	4
White	690 (28%)	11	620 (28%)	23
Residence				
Rural	499 (20%)	7	434 (20%)	16
Non-rural	1815 (74%)	6	1665 (75%)	15
Health insurance				
Uninsured	740 (30%)	3	553 (30%)	10
Insured	1712 (70%)	8	1310 (70%)	17
Time in HIV care, median (IQR)	9 (3, 17)	​	14 (7, 22)	​
0-4 years	782 (32%)	2	377 (17%)	3
≥5 years	1670 (68%)	8	1832 (83%)	17
HIV RNA level^e^				
≥200 copies/mL	492 (20%)	2	282 (4%)	6
<200 copies/mL	1927 (79%)	8	1910 (86%)	16
CD4 count, median (IQR)^e^	608 (377- 855)	​	633 (416- 864)	​
<250 cells/mm^3^	299 (12%)	5	237 (11%)	10
≥250 cells/mm^3^	2114 (86%)	7	1944 (88%)	15
Smoking history^e^				
Current	700 (29%)	12	578 (26%)	9
Former	300 (12%)	17	332 (15%)	16
Never	1452 (59%)	2	1299 (59%)	1
Smoking pack-years^d^, median (IQR)	15 (8-30)	​	15 (5-30)	​

^a^Eligible based on 2013 USPSTF guidelines for years 2018-2021, and 2021 guidelines for years 2022-2023.

^b^Measures are first available in each calendar period.

^c^Hispanic and other race is comprised of *n* = 288 Hispanic, *n* = 34 American Indian, *n* = 28 Asian, *n* = 89 self-identified as other.

^d^Only calculated among 1742 patients with a history of smoking.

^e^These measures may include values at care entry (CD4, HIV viral load, smoking status.)

### Lung cancer screening eligibility

**Figure 1.**
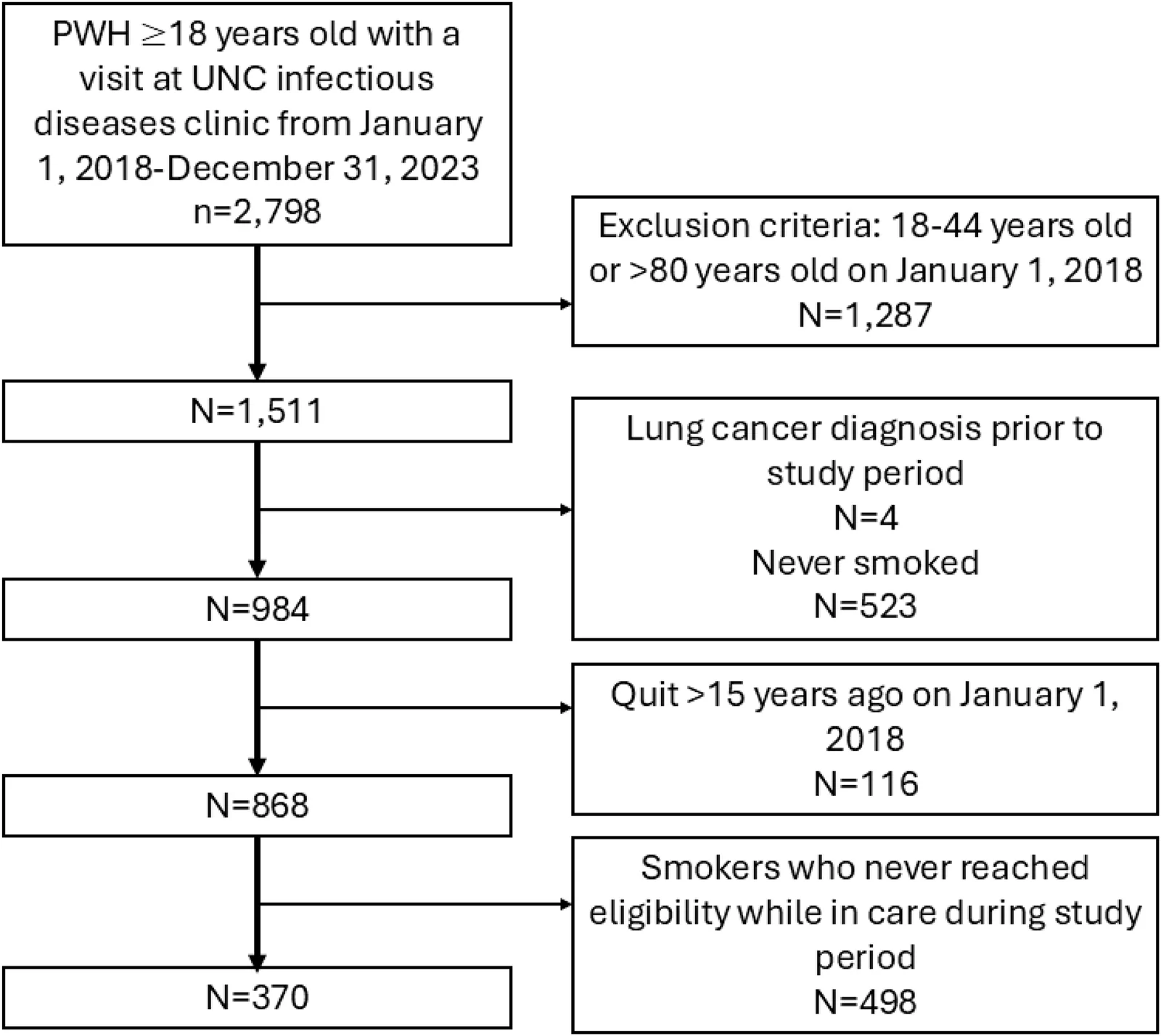
Lung cancer screening eligibility flowchart, 2018-2023.

From 2018 to 2023, 370 (13%) patients were eligible for LCS (Figure 1). A further 758 (44%) with a history of smoking did not meet the eligible age range of 50–80 years, and 627 (36%) did not meet the required pack-year smoking history. The annual proportion of patients eligible for screening remained constant at 6% from 2018 to 2021 and increased to 15% in both 2022 and 2023 (chi-square *p* < 0.05), corresponding to 2021 USPSTF guideline changes.

Differences in LCS eligibility were observed across patient demographic and clinical characteristics (Table 1). A smaller proportion of Black (5 and 14%) versus White (11 and 23%) patients were eligible for LCS in years 2018-2021 and 2022-2023, respectively. In contrast, PWH with 5 or more years in HIV care were more likely to be eligible for LCS compared to those with less than 5 years in care, as were patients with higher CD4 cell counts and suppressed HIV RNA levels.

### Lung cancer screening utilization

**Figure 2.**
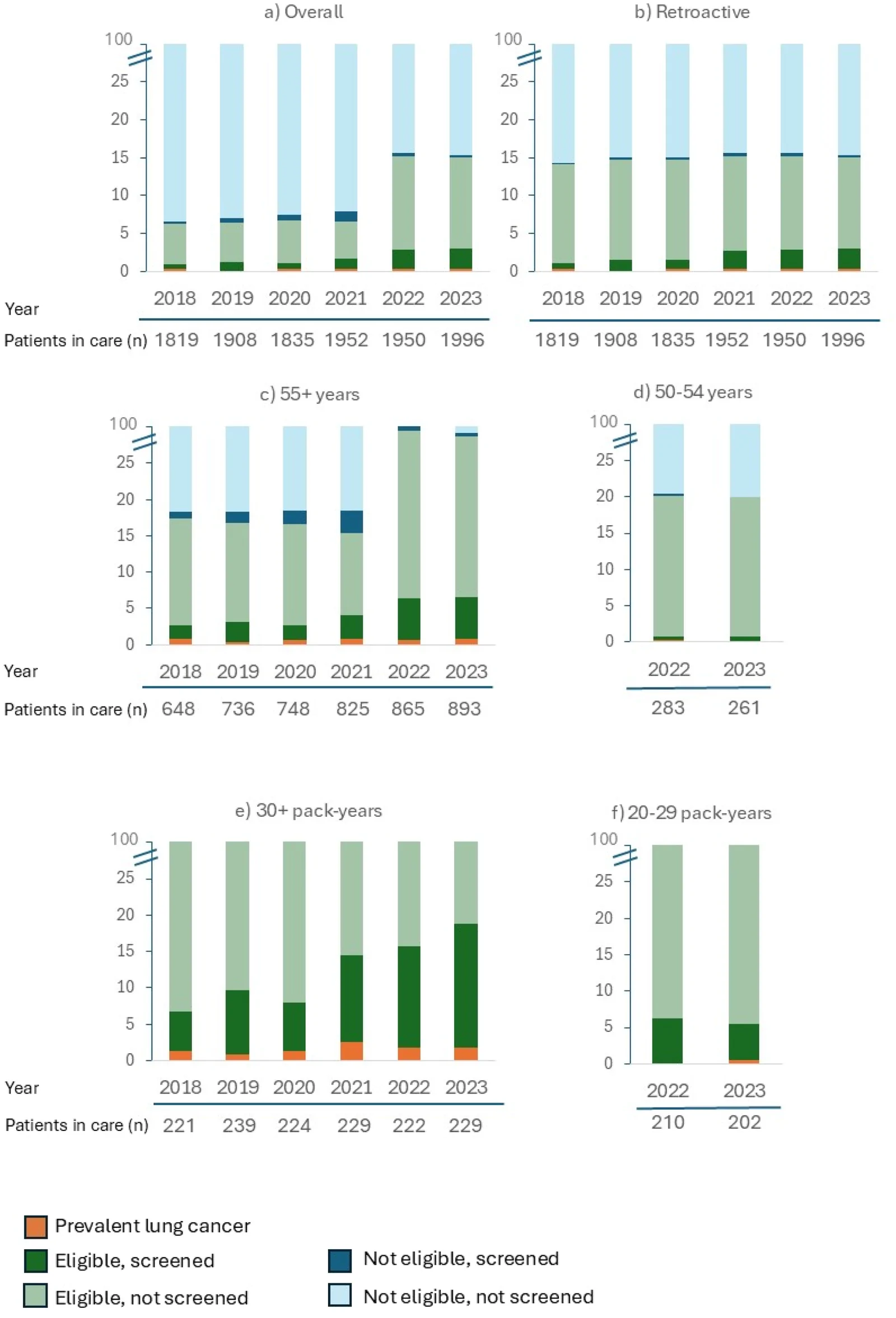
Lung cancer screening utilization by calendar year, 2018-2023. All patients with ≥1 visit at the UNC Infectious Disease Clinic were included in the total for that calendar year. *n = 2798*. Results reported overall including using updated guidelines in 2022-2023 (a); overall retroactively using new guidelines in all years (b); among patients at least 55 years old (c); 50–54 years old (new guidelines) (d); at least a 30 pack-year history (e); 20-29 pack-year history (new guidelines) (f).

Among 370 PWH eligible for LCS from 2018 to 2023, 101 (27%) received at least one LCS during the study period (Table 2). Annual screening utilization remained stable from 2018 to 2021 (ranging from 11 to 23%), increasing slightly in years 2022 and 2023 (18 and 24%, respectively). A modest, non-significant increase in screening was observed comparing 2018 (pre-guideline implementation) to 2023 (post-guideline implementation) of 11 and 24%, respectively; chi-square *p* = 0.10). We observed an initial increase then stability in the proportion of patients eligible for screening overall (Figure 2(a)), and when we implemented the 2021 USPSTF guidelines retroactively (Figure 2(b)). The modest increases in receiving LCS were observed when stratifying by age (Figure 2(c) and (d)) and pack-years of smoking (Figure 2(e) and (f)), as expected.

**Table 2.** Lung cancer screening with low dose computed tomography of the chest, 2018-2023.

Characteristic^b^	Overall (N, %)	≥1 LDCT^a^ (%)	Prevalence difference (%) (95% CI)
Overall	370	101 (27%)	​
Calendar year			
2018	108	12 (11%)	Ref.
2019	121	21 (17%)	6.2 (−2.7 - 15.2)
2020	119	15 (13%)	1.5 (−6.9 - 9.9)
2021	120	27 (23%)	11.4 (1.9 - 20.9)
2022	290	51 (18%)	6.5 (−0.9 - 13.8)
2023	294	54 (24%)	7.3 (−0.1 - 14.7)
**Age (years)**, median (IQR)	58 (55 - 62)	59 (57, 62)	​
50-59	239 (65%)	26%	Ref.
60-69	110 (30%)	32%	5.9 (−4.2 - 15.9)
70-80	21 (6%)	19%	−6.9 (−26.7 -12.9)
Sex			
Female	112 (30%)	30%	4.4 (−5.5 - 14.3)
Male	258 (70%)	26%	Ref.
Race/ethnicity			
Black	199 (54%)	31%	7.3 (−1.8 - 16.3)
Hispanic or other^c,d^	15 (4%)	6%	Not calculated
White	156 (42%)	25%	Ref.
Residence			
Non-rural	278 (75%)	26%	Ref.
Rural	92 (25%)	32%	5.6 (−4.9 - 16.1)
Health insurance			
Uninsured	58 (16%)	33%	6.4 (−6.0 - 18.9)
Insured	312 (84%)	26%	Ref.
Time in HIV care, median (IQR)	19 (12 - 24)	21 (12, 25)	​
0–4 years	21 (6%)	24%	Ref.
≥5 years	349 (94%)	28%	3.7 (−15.9 - 23.3)
HIV RNA level			
≥200 copies/mL	21 (6%)	19%	−8.9 (−28.5 - 10.8)
<200 copies/mL	344 (93%)	28%	Ref.
CD4 count, median (IQR)	656 (432, 870)	660 (434, 885)	​
<250 cells/mm^3^	30 (8%)	23%	−4.4 (−21.0 - 12.3)
≥250 cells/mm^3^	336 (90%)	28%	Ref.

Abbreviations. LDCT, low dose computed tomography; CI, confidence interval.

^a^LDCT estimated as having at least one LDCT during follow-up after eligibility.

^b^Time-varying factors measured at first lung cancer screening eligibility.

^c^Prevalence difference and 95% CI not estimated due to small sample size.

^d^Hispanic and other race is comprised of n = 7 Hispanic, *n* = 8 self-identified as other.

Among patients eligible for LCS, screening utilization was most prevalent in the 60-69 age group and least prevalent in the 70-80 age group. When both groups were compared to the 50-59 group, the differences in prevalence were not statistically significant (Table 2). There was an observed trend that patients with HIV RNA suppression were more likely to receive LCS (28 vs 19%; PD: −8.9, 95% CI: −28.5, 10.8), though this was not statistically significant.

## Conclusions

This study highlights both progress and persistent challenges in lung cancer screening among PWH. While the proportion of patients meeting LCS criteria increased steadily over time—coinciding with an aging population and the 2021 USPSTF expanded eligibility criteria—lung cancer screening uptake remained suboptimal, with less than a third of eligible PWH in care screened at least once. These findings represent a critical missed opportunity to detect early-stage lung cancers and reduce mortality in a population already burdened by high rates of tobacco use and HIV-related comorbidities. There is an urgent need to understand barriers to LCS uptake in order to implement targeted measures to reduce them, improve utilization and ultimately reduce lung cancer mortality in this high-risk population.

The 2021 USPSTF guidelines, which lowered the age threshold to 50 and the pack-year requirement to 20, corresponded with an increase in LCS eligibility. Approximately 15% of PWH seen at this large Southeastern HIV care center between 2022 and 2023 met eligibility guidelines for LCS, compared to 6% under previous guidelines. Although the proportion of patients eligible for screening increased over time and with the expansion of USPSTF recommendations, we did not observe increased utilization, with fewer than one-third of eligible patients receiving at least one LCS. This rate is comparable to that of the general US population, where screening uptake is estimated between 16 and 47%.^24,25,29^ Notably, the proportion of patients who met screening criteria but did not receive LCS increased following expansion of the recommendations. Our findings of missed opportunities for screening among those newly eligible with the 2021 recommendations, including those 50-55 years old and a 20-30 pack-year history, is particularly important as lung cancer in PWH presents at younger ages and with lower cumulative smoking history, making this group high risk and potentially with the most years of life to gain with early detection and intervention.^7–11^ This suggests that expanded eligibility alone is insufficient to drive meaningful improvements in LCS utilization in PWH; the persistent gaps likely reflect persistent individual, systemic, and structural barriers to screening.^23,30,31^

Improving LCS uptake among PWH will require addressing barriers unique to this population. Barriers to lung cancer screening uptake in the general population include individual factors such as financial burden and lack of knowledge, health system factors such as cost and complexity of scheduling, and social or environmental factors such as distance from clinic.^32^ A recent study among PWH indicated overall patient engagement with and enthusiasm for LCS; however, barriers to uptake included needing additional information, competing health care related and personal priorities, and economic concerns.^33^ These factors, combined with the burden of HIV management and associated comorbidities, may create competing priorities for both patients and providers that hinder adherence to cancer screening recommendations. As recommended laboratory monitoring intervals have lengthened for many PWH on stable ART, clinic visits have become less frequent, reducing opportunities to address preventative health needs.^34^ Similarly, many PWH have not established primary care outside of HIV care due to stigma, limited access, or insurance barriers, further limiting opportunities to implement LCS. Potential implementation strategies that have been evaluated in the general population include decision aids, shared decision-making tools, and scheduling support for patients; however, these have not been evaluated in the unique setting where PWH receive care.^35–37^ There is a critical need to characterize the complexity of multi-level barriers using an implementation science framework in order to identify potential interventions to improve screening uptake, minimize risks of screening, and ultimately improve cancer-related mortality in PWH.

This study has several limitations, including limited ability to disentangle the impact of the COVID-19 pandemic on screening utilization during this period, though evidence suggests that screening utilization did not change dramatically from 2019 to 2020.^38^ Our cross-sectional study design allowed us to estimate annual prevalence of LCS eligibility and utilization in the clinic population in each calendar year; however, this approach restricts us from inferring changes within individuals longitudinally. We relied on CPT and historical HCPCS codes and may have missed LCS billed under alternative codes. This analysis was limited to a single-center tertiary care ID clinic; larger, multicenter studies are needed to improve generalizability and better account for variation in clinical context. However, our study population in the Southeastern U.S. includes a high burden of tobacco use and a substantial rural population. Given the high proportion of individuals at elevated lung cancer risk in this population, we expect that these findings are an important step toward understanding gaps in LCS utilization among a particularly vulnerable group.

The 2021 USPSTF guideline update expanded LCS eligibility among PWH, but this expansion has not translated into substantial increases in screening. Improving uptake of guideline-indicated LCS will require a multifaceted approach that integrates LCS into routine HIV care with a focus on engaging underserved populations. Lung cancer screening decision aids are being adapted for use among PWH,^39^ and, together with tailored interventions such as patient education and system-level reminders, may be critical to closing the screening gap and improving lung cancer outcomes in this high-risk population.
